# Social support-based physical activity that exerts beneficial effects for obese older adults with cognitive impairment via increasing participation in leisure-time physical activity

**DOI:** 10.1371/journal.pone.0325516

**Published:** 2025-06-30

**Authors:** Savitree Thummasorn, Piangkwan Sa-Nguanmoo, Pornpen Sirisattayawong, Akleema Ingding, Chanakarn Kumkun, Nitchakarn Pittayaporn, Patcharawalai Tupsai, Sasinart Morthin, Orawan Phithakleelanon

**Affiliations:** 1 Department of Occupational Therapy, Faculty of Associated Medical Sciences, Chiang Mai University, Chiang Mai, Thailand; 2 Department of Physical Therapy, Faculty of Associated Medical Sciences, Chiang Mai University, Chiang Mai, Thailand; Taipei Medical University, TAIWAN

## Abstract

Obesity in older adults increases the risks of diabetes, cardiovascular disease, cognitive decline, and depression. Physical activity has been established as an important strategy in lifestyle interventions for obese people. This study focused on the strategy for increasing participation in physical activity, which is called social support. Although the studies found that social support helps to motivate participation in physical activity of older adults, its beneficial effects for population with obesity are still few investigations. Therefore, this study focused on developing a social support-based physical activity program for obese older adults with cognitive impairment and hypothesized that it can increase level of physical activity and reduce severity of depression and cognitive decline in obese older adults with cognitive impairment. In this study, thirty-nine participants were divided into three groups consisting of 13 obese older adults, 13 obese older adults with cognitive impairment, and 13 obese older adults with cognitive impairment and receiving the physical activity program for 4 weeks. After that, the levels of physical activity, cognition, and depression were tested in all three groups. Results showed that social support-based physical activity decreased cognitive decline and severity of depression in obese older adults with cognitive impairment. Moreover, the level of physical activity in obese older adults with cognitive impairment and receiving the physical activity program was increased by increasing participation in leisure-time physical activity. Thus, it suggested that the social support-based physical activity exerted beneficial effects for obese older adults with cognitive impairment via increasing participation in leisure-time physical activity.

## Introduction

Nowadays, the prevalence of obesity in older adults is rising progressively [1]. Other studies have reported that obesity in older adults increases the mortality rate as well as the risks of diseases such as diabetes, metabolic syndrome, cardiovascular disease, hypertension, etc. [2–4]. Moreover, obesity increases the risk of cognitive decline in older adults [5,6], which leads to the progression of mild cognitive impairment as well as dementia [7]. Interestingly, those studies also found that obesity correlated with the risk of depression, anxiety, and stress [8,9]. Therefore, the researchers tried to prevent and reduce these adverse effects in obese older adults by using strategies of intervention such as diet control, exercise, and increasing metabolism.

Recently, the approach for clinical management in obese older adults can be conducted in health professionals via promoting appropriate nutrition, adapting behaviors, and increasing levels of metabolism and physical fitness [10–12]. This study focuses on an approach of lifestyle modification via increasing physical activity in the obese population. It has become known that physical activity is an important strategy in lifestyle interventions for obese people [13], and study has shown that an increase of participation in physical activity can improve mental and physical functions in obese older adults [14]. Moreover, it can prevent and reduce the risks of cognitive impairment and dementia in older adults [15,16]. Interestingly, evidence has suggested that an increased participation in physical activity can reduce the risks of depressive symptoms in older adults with mild to moderate depression [17,18]. Thus, increased participation in physical activity by using lifestyle modification probably reduces cognitive impairment and severity of depression in obese older adults.

This study focused on the strategy for increasing a participation in physical activity, which is called social support [19]. It is known that social support such as family, loved ones, or friends forms a social environment that can change behavior regarding health in an individual [20]. Moreover, evidence has suggested that social support correlates with an increased ability to perform the leisure-time physical activity in older adults [21]. Social support can increase a participation of individuals in physical activity via increasing observational learning, sharing experiences, and increasing motivation [19]. Therefore, the World Health Organization (WHO) suggested that social support is a key determinant of active ageing as well as maintaining quality of life for older adults [22]. Interestingly, a study reported that good social support also reduced the risk of depression [23] and cognitive decline in that population [24]. Thus, it is possible that modifying the social environment, by using the concept of social support, may increase engagement in lifestyle activities, and lead to a decrease of cognitive decline and depression in obese older adults. Although the previous studies suggested that social support helps to motivate a participation in physical activity of older adults [25,26], its beneficial effects in population with obesity and obese people with cognitive impairment are still few investigations. Therefore, this study focused on developing a social support-based physical activity program for obese older adults with cognitive impairment and hypothesized that it can increase level of physical activity and reduce severity of depression and cognitive decline in obese older adults with cognitive impairment. Furthermore, the information provided an understanding of the beneficial effects of a social support approach for increasing participation in physical activity in obese older adults with cognitive impairment.

## Materials and methods

### Research design and participants

**Fig 1:**
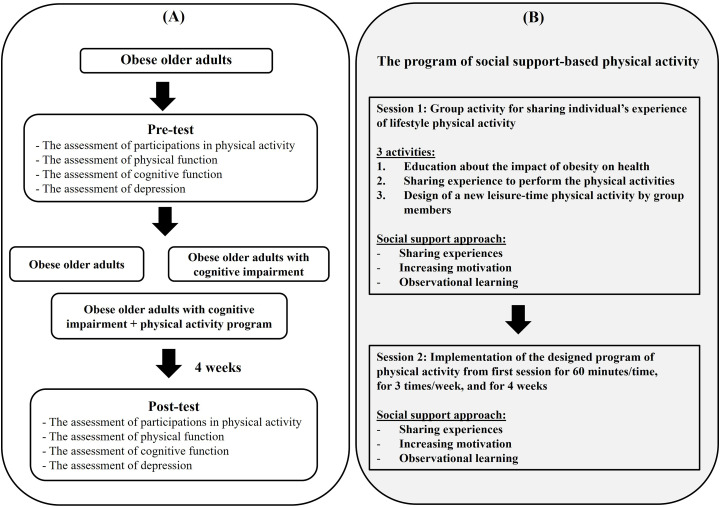
A. The research design of this study, B. The social support-based physical activity program.

Ethics approval was provided by the Ethics Committee in accordance with relevant guidelines and regulations (Ethic number: AMSEC-64EX-128). All of the participants provided written informed consent. The sample size was based on a previous study, in which 13 participants took part, and the results showed that walking exercise can increase energy and physical function in obese adults [27]. Then, a sample size calculation was conducted using G*Power software 3.1.9.7 (effect size = 0.9, power of test = 0.7, alpha = 0.05). The 39 participants (aged older than 60 years) in this study were divided into three groups consisting of 13 obese older adults, 13 obese older adults with cognitive impairment, and 13 obese older adults with cognitive impairment and receiving a four-week physical activity program. All of the data in this study were collected from the community-dwelling of older adults in Ban Rong Wua Health Promoting Hospital and Ban Sai Mun Health Promoting Hospital, San Pa Tong district, Chiang Mai province, Thailand, from April 2022 to July 2022. Body mass index (BMI) was established as an indicator for obesity. In classifications of the Asia-Pacific body mass index, BMI ≥ 25 kg/m^2^ was the cutoff point of obesity in the Asian population [28,29]. Thus, BMI ≥ 25 kg/m^2^ was used for screening obesity in this study. Additionally, the Mini-Mental State Examination (MMSE) was used in this study for screening cognitive function between obese older adults and those with cognitive impairment [30]. The eligibility criteria required in this study is that participants do not have psychiatric diagnosis and physical disability. Then, the outcomes of this study, including participation in physical activity, physical function, cognitive function, and depression level, were investigated at the baseline (pre-test) and end of physical activity program (post-test) on the same day in all of the groups. The research design of this study is shown in Fig 1A.

### Design of physical activity program

It is known that the domains of physical activity are divided into four domains including household, transportation, occupational, and leisure-time [31]. This study focuses on developing and studying the effects of leisure-time physical activity on health in population with obesity. The studies found that leisure-time physical activity has beneficial effects for improvement of health and decrease in risk of cardiovascular mortality [31,32]. Additionally, evidence has suggested that social support which is a strategy for changing the health behavior of an individual, correlates with an increased ability to perform the leisure-time physical activity in older adults [21]. Moreover, social support can increase positive health outcomes via shaping health behavior in population with obesity [33]. So, the program for leisure-time physical activity for obese older adults with cognitive impairment was developed based on the concept of social support in this study. This study designed the program by using social groups that have the same health problem (obesity) and same interest in physical activity, as in social support. It was hypothesized that a social group could encourage and increase participation in the physical activity of its members via increasing motivation, sharing experiences, and increasing observational learning.

The program of leisure-time physical activity by using social groups consisted of two sessions as follows. First, the members were encouraged to participate in a focus group. A focus group was conducted by occupational therapists for sharing experiences of leisure-time physical activity of group members. Three activities in the first session were performed for two hours included: 1) learning from occupational therapists of how obesity impacts health, 2) sharing experiences of performing physical activities in a group, and 3) designing specific leisure-time physical activity by members within a group, while under the supervision of occupational therapists. The aim of this session is to encourage the participants to design their own movement with therapists. The details of elements of dance are shown in Table 1.

**Table 1:** The details of elements of dance.

Patterns of dance	Body part and movements	Direction of movements	Intensity level	Rhythm of music
**Stretching exercise** **(10 minutes)**	Neck stretch Arm and shoulder stretch Hip flexors stretch Leg stretch Ankles and toes stretch	Forward/backward, right/left, up/down	High to middle	Regular rhythm
**Thai folk dance** **(40 minutes)**	**Body parts:** Head, shoulders, arms, pelvis, trunk, legs, knees, and ankles **Movements:** Slow walk, speed walk, sitting, stand-up Pull, push, bend, stretch, and swing	Forward/backward, right/left, up/down	High to middle	Alternating rhythm Progressive rhythm Flowing rhythm
**Cool down exercise** **(10 minutes)**	Neck stretch Arm and shoulder stretch Back stretch Leg stretch Ankles and toes stretch	Forward/backward, right/left	Middle to low	Regular rhythm

In the next step, the content validity of the developed program was performed by five experts consisting of a doctor, two occupational therapists, and two physical therapists. In this section, a discussion panel by experts was conducted on topics: 1) appropriateness of patterns of dance, movements, intensity level, direction of movements, and rhythm of music, 2) appropriateness of frequency and duration for performing activity in obese older adults, 3) analyze the risks may arise during activity. Finally, the program of the social support-based physical activity was modified based on experts’ suggestions before the implementation.

Second section, the program of physical activity designed from the first session was implemented for 60 minutes in obese older adults with cognitive impairment, three times per week for four weeks. The social support-based physical activity program is shown in Fig 1B.

### Assessment of study

#### Assessment of participation in physical activity.

The Global Physical Activity Questionnaire (GPAQ) was used as a tool for assessing physical activity participation in three domains, including physical activity at work, transport-related physical activity, and recreational activity or leisure-time physical activity [34].

#### Assessment of physical function.

The 6-minute walk test (6-MWT) was used for assessing mobility-related functioning and endurance in obese older adults via investigating arterial oxygen saturation (SPO_2_), heart rate, and distance walked on a flat/hard surface in a period of 6 minutes [35].

#### Assessment of cognitive function.

The Montreal Cognitive Assessment (MoCA) was used to assess cognitive impairments in older adults [36]. Its score of 25 or above is considered as normal cognition.

#### Assessment of depression.

The severity of depressive symptoms was measured by using the Thai version of the Patient Health Questionnaire (PHQ-9) [37]. Its total scores can be interpreted as the level of depressive symptoms as follows: normal level (PHQ-9 score from 0 to 4 points), mild depressive level (PHQ-9 score from 5 to 9 points), moderate depressive level (PHQ-9 score from 10 to 14 points), moderately severe depression level (PHQ-9 score from 15 to 19 points), and severe depressive level (PHQ-9 score from 20 to 27 points) [37]. In this study, the PHQ-9 scores represented the level of depression in each group.

#### Data analysis.

All of the data were presented as mean values ± SD. The Pearson χ2 test was used to analyze categorical variables, which were expressed as frequencies. In this study, the one-way ANOVA was used to compare the levels of physical activity, depression, and cognition among three groups. The homogeneity of variance was assessed using Levene’s test for equality of variances. After performing the homogeneity of variance and ANOVA, the means of each group were compared by using a post hoc test (Bonferroni). The Bonferroni correction is used for adjusting the significance level that can occur in multiple comparisons. It is known that this method can protect the type I error rate of data [38]. Additionally, a dependent t-test was used to compare the average scores of all parameters between pre- and post-test within the same group [39]. T-test is used when the variables are normally distributed. A *P* value < 0.05 was statistically significant.

## Results

### General characteristics of the participants

General characteristics of the participants in the three groups are shown in Table 2. In this study, the parameters of general characteristics tested included gender, age, BMI, education, underlying disease, and history of head injuries. The results showed that all of the parameters showed no significant difference among the three groups.

**Table 2:** General characteristics of participants.

General characteristics	Obese older adults (n = 13)	Obese older adults with cognitive impairment (n = 13)	Obese older adults with cognitive impairment and receiving physical activity program (n = 13)	Sig.
**Gender (Frequency)** Male/Female	4/9	5/8	5/8	No
**Age (years)** Average (SD)	64.23 ± 1.83	63.62 ± 3.31	64.00 ± 1.41	No
**BMI (kg/m**^**2**^**)** Average (SD)	27.31 ± 1.60	26.69 ± 1.03	26.31 ± 1.11	No
**Education (Frequency)** Uneducated/ Educated	0/13	0/13	0/13	No
**Underlying disease (Frequency)** Yes/No	12/1	12/1	11/2	No
**History of head injuries (Frequency)** Yes/No	0/13	0/13	0/13	No

BMI: Body mass index.

* p < 0.05 vs. Obese older adults, # p < 0.05 vs. Obese older adults with cognitive impairment.

### Participation in physical activity

**Fig 2:**
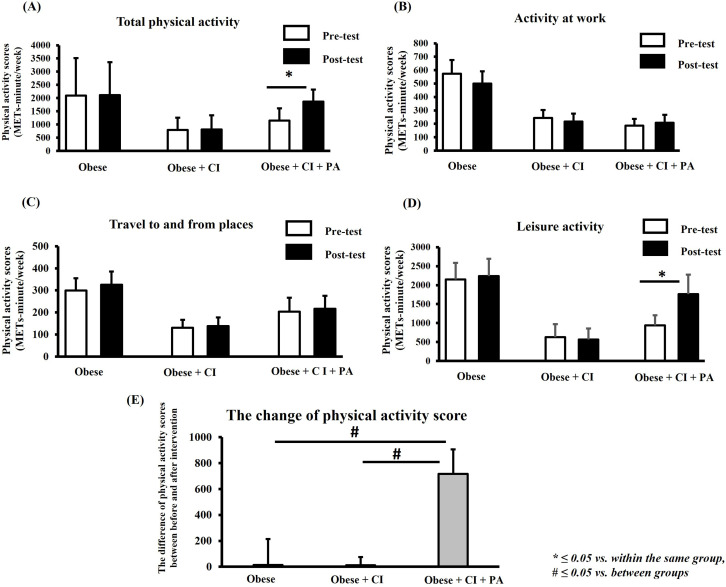
The participation in physical activity. The total score of physical activity (A), average score of physical activities at work (B), average score of transportation activity (C), and average score of leisure-time physical activity (D) between the pre- and post-test within the same group. The difference of total physical activity scores among the groups (E). * ≤ 0.05 vs. within the same group, # ≤ 0.05 vs. between groups. CI = cognitive impairment, PA = physical activity.

Participation in physical activity between pre- and post-test within the same group is shown in Fig 2A–D. Fig 2A represents participation in total physical activity, and results showed that its average score had increased significantly post-test in a group receiving physical activity program, when compared to that pre-test. However, this study found that the scores of physical activities in work and transportation domains were not significantly different between pre-and post-test in any of the groups (Fig 2B, C). Interestingly, the result showed that the score of leisure-time physical activity increased significantly post-test in group receiving physical activity program, when compared to the that pre-test (Fig 2D). In addition, this study also compared the change of scores in total physical activities among the groups (Fig 2E). In this study, the difference in total physical activity scores between pre- and post-test is represented by the change of scores in physical activities in each group. The results showed that the level of total physical activity in the group receiving physical activity program was increased significantly when compared to that in control group and group without physical activity program (Fig 2E).

### The physical function

**Fig 3:**
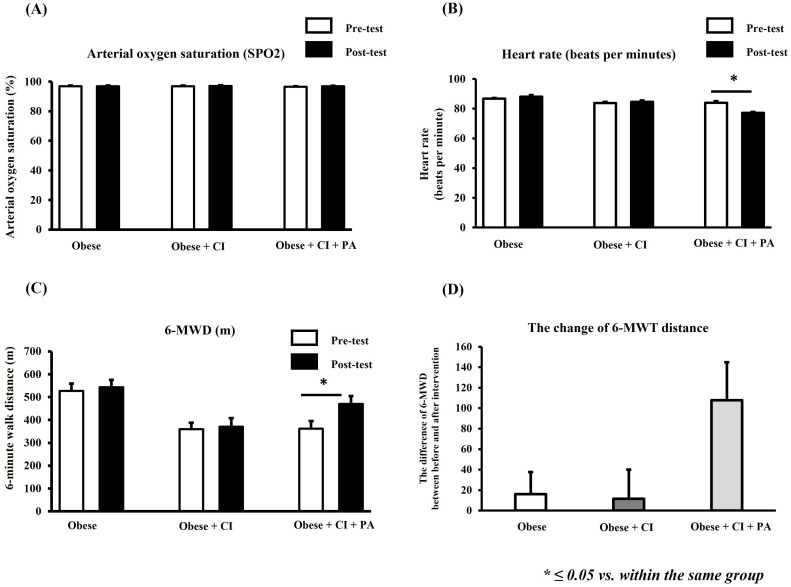
The physical functional capacity. The results of physical functioning included arterial oxygen saturation (SPO2) (A), heart rate (B), and 6-MWT distance (6-MWD) (C) between the pre- and post-test within the same group. The difference of 6-MWD among the groups (D). * ≤ 0.05 vs. within the same group. 6-MWD = 6-minute walk distance, CI = cognitive impairment, PA = physical activity.

The 6-MWT was used to assess physical functional capacity in obese older adults, and its results including SPO_2_, heart rate, and 6-MWD, are shown in Fig 3A–C. The results in this study showed that SPO_2_ within the same group was not significantly different between pre- and post-test in any of the groups (Fig 3A). However, this study found that the heart rate was significantly decreased post-test in a group receiving physical activity program, when compared with that pre-test (Fig 3B). Moreover, the 6-MWD was increased significantly by post-test in a group receiving physical activity program, when compared to that pre-test (Fig 3C). Furthermore, this study also compared the change of 6-MWD among the groups (Fig 3D). In this study, the difference in 6-MWD between pre- and post-test is represented by the change of 6-MWD in each group. The results in this study showed that the change of 6-MWD showed not significantly different among groups (Fig 3D).

### The cognitive function

**Fig 4:**
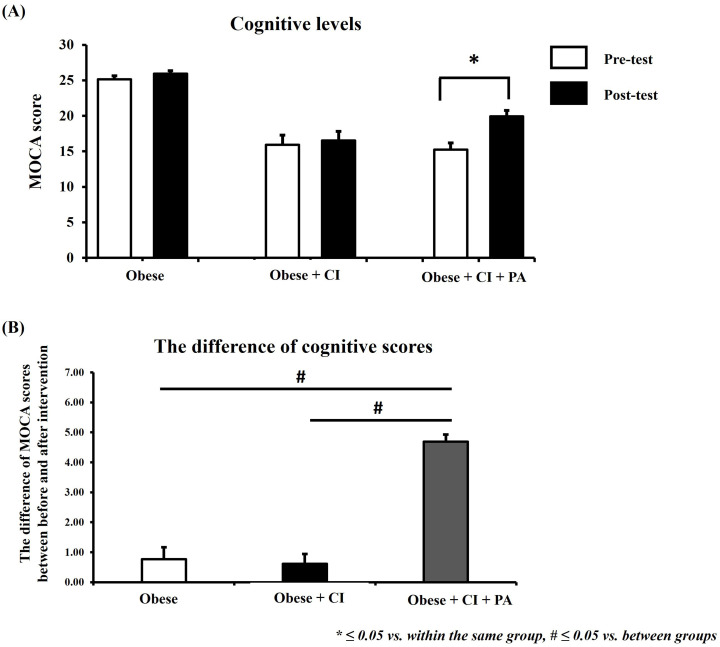
The level of cognitive function. The MoCA scores between the pre- and post-test within the same group (A). The difference of MoCA score among the groups (B). * ≤ 0.05 vs. within the same group, # ≤ 0.05 vs. between groups. MoCA = Montreal Cognitive Assessment, CI = cognitive impairment, PA = physical activity.

The cognitive function is shown in Fig 4A–B. The increase in MoCA score correlates with a decrease of cognitive decline. Our results demonstrated that the MoCA score increased significantly post-test in a group receiving physical activity program, when compared to that pre-test (Fig 4A). In addition, this study also compared the change of MoCA score among the groups (Fig 4B). In this study, the difference of MoCA score between pre- and post-test is represented by the change of cognitive scores in each group. The results of this study showed that the MoCA score was increased significantly in a group receiving physical activity program when compared to that in control group and group without physical activity program (Fig 4B).

### The depression level

**Fig 5:**
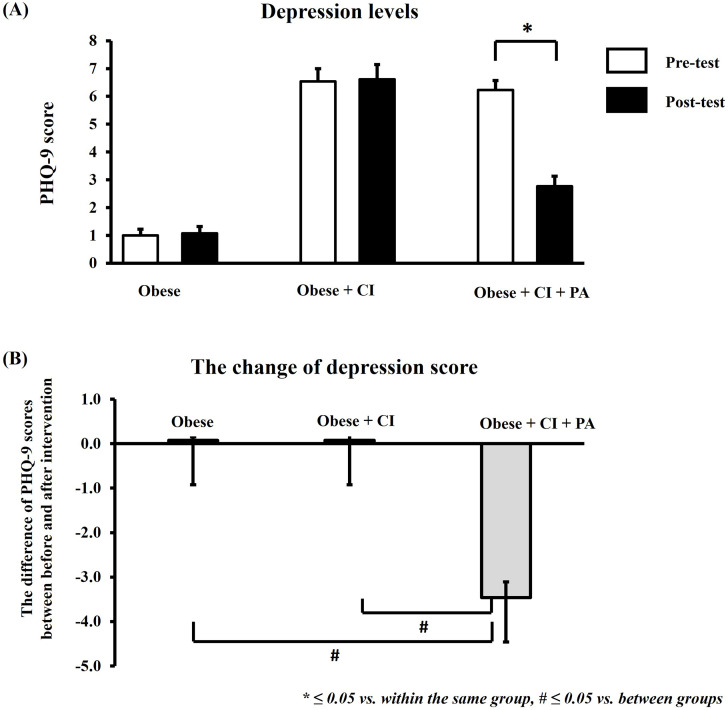
The level of depression. The PHQ-9 scores between the pre- and post-test within the same group (A). The difference of PHQ-9 scores among the groups (B). * ≤ 0.05 vs. within the same group, # ≤ 0.05 vs. between groups. PHQ-9 = Patient Health Questionnaire-9, CI = cognitive impairment, PA = physical activity.

In this study, the depression score was determined by using the PHQ-9. The decrease in PHQ-9 score correlated with reduced severity of depression. The result showed that the PHQ-9 score in post-test decreased significantly in a group receiving physical activity program, when compared to that pre-test (Fig 5A). Furthermore, this study also compared the difference of PHQ-9 score among the groups (Fig 5B). In this study, the difference in PHQ-9 scores between pre- and post-test is represented by the change of depression scores of each group. These results showed that the PHQ-9 score decreased significantly in a group receiving physical activity program when compared to that in control group and group without physical activity program (Fig 5B).

## Discussion

It is established that obesity was positively associated with cognitive impairment in middle-aged and older adults [40]. However, our result of Fig 4B found that a change of cognitive scores did not different between the obese group and obese group with cognitive impairment. There are two possibilities for this finding. First, the severity of obesity in this study may accelerate cognitive decline in older adults. Thus, the level of cognitive impairment in obese older adults with cognitive impairment seems not to change when compared with obese older adults alone. It is consistent to a previous study reported that obesity can accelerate cognitive decline in older adults [26]. Second possibility, levels of BMI may be required up to a critical level for triggering cognitive decline. When the BMI levels reached the level of BMI critical threshold, the cognitive decline can be observed. This possibility is consistent to the study demonstrated that BMI is positively associated with cognitive levels for BMI up to the threshold, and then the relationship turns negative for BMI greater than the threshold [41]. Therefore, the cognitive level in this study did not differ between the obese group and obese group with cognitive impairment. However, this hypothesis needs to be tested in the future.

**Fig 6:**
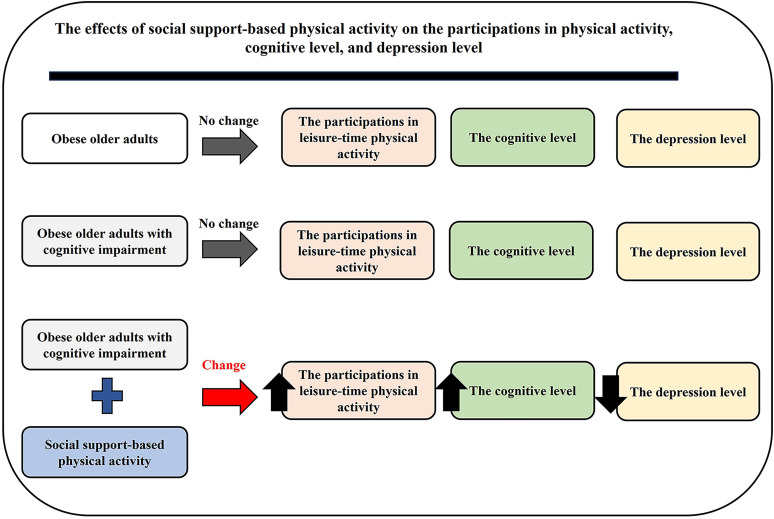
A summary of the findings of study.

Additionally, it is demonstrated that an increase in cognitive impairment of older adults can lead to the risk of depression and a decrease in physical activity [42,43]. However, our results showed that the levels of physical activity and depression in the obese group were not significantly different when compared to the obese group with cognitive impairment (Figs 2E and 5B). It is possible that the level of cognitive decline in the obese group may reach to the level of critical threshold for triggering the changes of physical activity and depression. Therefore, the levels of physical activity and depression did not differ between the obese group and obese group with cognitive impairment. In the future, it needs to investigate this hypothesis. After intervention, the results demonstrated that the social support-based physical activity program could reduce cognitive decline and severity of depression in obese older adults with cognitive impairment. Moreover, social support-based physical activity also increased participation in physical activity for such adults, especially by increasing participation in leisure-time physical activity. A summary of the findings of the study is shown in Fig 6.

Studies suggested that ageing is associated with changes in metabolism, which leads to the risks of obesity, insulin resistance and metabolic syndrome in older adults [44,45]. Especially, the change in body composition and fat distribution is important key for an increased BMI in older adults [46]. Then, these changes can lead to the risks of obesity in older adults. Evidence showed that obesity decreased cognitive performance as well as accelerated cognitive decline in such people [26]. Moreover, obesity is associated with increased risks of depression and mental health problems [8,9]. Thus, the prevention and decrease of obesity may reduce cognitive decline and severe depression, which would lead to maintaining quality of life for obese older adults. In clinical practice, occupational therapists play role as health management by increasing participation in activities of choice and through lifestyle interventions in obese population [47]. Moreover, occupational therapists can also encourage health behavior and well-being in obese older adults by promoting occupational balance and appropriate social support [47]. After that, researchers are focusing on finding new interventions for preventing risks of obesity in older adults.

It became known recently that physical activity is an important strategy for lifestyle interventions in the obese population [10]. Study showed that increased participation in physical activity can improve mental and physical functions for these people [11]. In occupational therapy, participation in physical activity can be conducted in four dimensions including assessment, education, environmental modification, and adaptation to occupations [48]. This study developed a new program of physical activity based on the concept of social support for such individuals. Social support is an environmental factor that increases motivation and participation of the individual in performing occupations [49]. Moreover, studies have reported that social support is a strategy for maintaining and adapting health behavior in older adults [50]. Thus, adopting social support may change health behavior for the better in obese older adults with cognitive impairment. Examples of a social environment for individuals are friends, family, neighbors, people living in the same community, etc. This study designed membership within the group with the opportunity of performing physical activities together. The criteria of social support in this study were members within groups living in the same community, having obesity, and having interest in performing similar physical activities. The results showed that the social support-based physical activity program increased physical functional capacity and participation in physical activity for obese older adults with cognitive impairment. These findings were consistent with another study reporting that performing sufficient physical activity is a healthcare strategy, which can increase physical health benefits in older adults [21]. Engaging in social support-based physical activity reduced post 6-MWT heart rate. Participating in physical activity may be ascribed to the comprehensive involvement of the respiratory, circulatory, and muscular systems [51]. Consequently, our physical activity program can increase physical functional capacity. Moreover, the findings in this study showed that the social support-based physical activity program increased the level of leisure-time physical activity. It is possible that social support in our physical activity program increases the level of total physical activity in obese older adults with cognitive impairment via increasing participation in leisure-time physical activity. This is consistent with a study suggesting that good social support increases participation in leisure-time physical activity via increasing observational learning, experience sharing, and increased motivation in women having breast cancer treatment [19].

Additionally, the results in this study showed that social support-based physical activity reduced cognitive decline in obese older adults with cognitive impairment, and there are two possibilities for this. First, our program of social support-based physical activity reduced cognitive decline via modifying the social environment of individuals. This is consistent with a study reporting that good social support correlated with reduction of cognitive decline in older adults [52]. Second, increased participation in physical activity, induced by our program, could reduce cognitive decline in obese older adults with cognitive impairment. This hypothesis is consistent with a study reporting that physical activity programs increased cognitive and metacognitive functions in children and adolescents [53]. In addition, a previous study found higher levels of physical activity and improved functional capacity are linked to the preservation of cognitive function [54]. In future, the mechanism of social support-based physical activity program for reducing cognitive decline in obese older adults with cognitive impairment needs to be studied.

In addition, the results in this study showed that cognitive impairment increased the risks of depression in obese older adults. It is possible that the lack of social support for older adults may decrease the opportunity of social participation, which leads to mental health problems such as depression. This hypothesis is consistent with studies reporting that retirement, which is a life event for older adults, increases the level of loneliness and social isolation [55,56]. Moreover, the results in this study showed that the social support-based physical activity program can reduce the severity of depression in obese older adults with cognitive impairment. It is possible that increasing social interaction via group activity has the beneficial effect of reducing the severity of depression in such people. Therefore, this study suggested that the physical activity program, induced by social support, can reduce the severity of depression in obese older adults with cognitive impairment. The mechanism of the social support-based physical activity program for reducing the level of depression needs to be tested in the future.

Although many studies have demonstrated that group-based exercise can improve physical and cognitive functions in older adults with cognitive impairment [57,58], the effect of group-based physical activity has never been investigated in obese older adults with or without cognitive impairment. Thus, this study developed a program of physical activity based on the concept of social support for such individuals. The concept of this study focuses on encouraging individuals to participate in designing their own movement. Moreover, giving opportunities for individuals to share and discuss their experiences of performing physical activities within a group is the highlight of this intervention program. A perspective of this study believes that sharing experiences of performing physical activities within a group via focus group could encourage and increase participation in the physical activity of group members via increasing motivation, sharing experiences, and increasing observational learning. Moreover, increased participation in the physical activity of individuals via social support is an important strategy for maintaining and adapting health behavior of individuals. Therefore, this study suggested that social support-based physical activity may have potential effects for obese older adults with cognitive impairment via increasing participation in leisure-time physical activity. All information from this study can provide an understanding of the beneficial effects of an intervention strategy for obese older adults with cognitive impairment.

## Study limitations

Overall, social support-based physical activity may have potential effects for obese older adults with cognitive impairment via increasing participation in leisure-time physical activity. However, there are two limitations of this study. First, because all outcomes of this study were not different between the obese older adults alone and obese older adults with cognitive impairment. Thus, it is the absence of a direct comparison group (obese older adults with cognitive impairment not receiving the intervention) in this study. It is possibility that the severity of obesity in this study may accelerate cognitive decline in older adults. Therefore, the level of cognitive impairment in obese older adults with cognitive impairment was not to change when compared with obese older adults alone. In the future, this possibility needs to be tested. Second limitation, it is not possible to definitively attribute the observed improvement solely to intervention. Other factors, such as natural changes over time, the attention received by participants (Hawthorne effect), or expectations of improvement (placebo effect), could have contributed to the results. In the future, these possibilities should be also tested.

## Conclusion

This study demonstrated that social support-based physical activity decreased cognitive decline and severity of depression in obese older adults with cognitive impairment. Moreover, it also increased physical performance and the level of participation in physical activity in such people through increasing ability to perform leisure activities. Therefore, this study suggested that social support-based physical activity may have potential effects for obese older adults with cognitive impairment via increasing participation in leisure-time physical activity.

## Supporting information

S1 Fig Participation in physical activity. The total score of physical activity (A), average score of physical activities at work (B), average score of transportation activity (C), and average score of leisure-time physical activity (D) between the pre- and post-test within the same group. The difference of total physical activity scores among the groups (E). The data set is shown below.
(PDF)

S2 Fig The physical functional capacity. The results of physical functioning included arterial oxygen saturation (SPO2) (A), heart rate (B), and 6-MWT distance (6-MWD) (C) between the pre- and post-test within the same group. The difference of 6-MWD among the groups (D). The data set is shown below.
(PDF)

S3 Fig The level of cognitive function. The MoCA scores between the pre- and post-test within the same group (A). The difference of MoCA score among the groups (B). The data set is shown below.
(PDF)

S4 Fig The level of depression. The PHQ-9 scores between the pre- and post-test within the same group (A). The difference of PHQ-9 scores among the groups (B). The data set is shown below.
(PDF)
