# Supplementary material for: Social support-based physical activity that exerts beneficial effects for obese older adults with cognitive impairment via increasing participation in leisure-time physical activity
Source: PLoS One. 2025 Jun 30;20(6):e0325516. doi: 10.1371/journal.pone.0325516 (PMC12208442; doi:10.1371/journal.pone.0325516)
Supplement: S1 Fig — The total score of physical activity (A), average score of physical activities at work (B), average score of transportation activity (C), and average score of leisure-time physical activity (D) between the pre- and post-test within the same group. The difference of total physical activity scores among the groups (E). The data set is shown below. (PDF) [file pone.0325516.s001.pdf]

S1 Fig. Participation in physical activity.

The total score of physical activity (A), average score of physical activities at work (B), average score of transportation activity (C), and average score of leisure-time physical activity (D) between the pre- and post-test within the same group. The difference of total physical activity scores among the groups (E).

The total score of physical activity (A)

The total score of physical activity (A)					
Obese		Obese + CI		Obese + CI + PA	
Pre	Post	Pre	Post	Pre	Post
1340	1440	1700	1880	1200	1980
5520	3820	1200	1520	720	2000
600	720	920	760	480	2160
1760	3360	440	660	480	2100
1220	1240	360	400	900	2520
1200	1320	1200	1120	1280	1200
2240	2760	520	360	980	1740
1580	1330	200	440	1380	2020
4340	4720	720	180	2120	2400
1460	1520	480	480	1240	1020
1560	1260	720	720	1780	1680
1200	1080	430	420	1180	2100
3200	2840	1450	1560	1150	1280

	Obese		Obese + CI		Obese + CI + PA	
	Pre	Post	Pre	Post	Pre	Post
**Mean**	**2093.85**	**2108.46**	**795.38**	**807.69**	**1145.38**	**1861.54**
**SD**	1421.63	1250.20	463.71	540.53	464.84	457.93
**SE**	394.289	346.743	128.610	149.915	128.924	127.006

Average score of physical activities at work (B)

The average score of physical activities at work (B)					
Obese		Obese + CI		Obese + CI + PA	
Pre	Post	Pre	Post	Pre	Post
500	400	480	400	0	0
900	700	60	80	160	240
450	360	50	0	0	0
720	650	480	500	200	280
260	160	100	200	420	500
360	450	200	120	0	0
0	0	160	0	240	240
952	752	240	200	260	360
1040	1140	60	180	350	240
480	580	0	240	200	160
720	420	720	0	0	0
0	0	260	180	580	680
1080	880	360	720	0	0

	Obese		Obese + CI		Obese + CI + PA	
	Pre	Post	Pre	Post	Pre	Post
**Mean**	574.00	499.38	243.85	216.92	185.38	207.69
**SD**	364.92	334.23	213.37	212.11	186.93	215.64
**SE**	101.211	92.698	59.177	58.828	51.846	59.809

Average score of transportation activity (C)

The average score of transportation activity (C)					
Obese		Obese + CI		Obese + CI + PA	
Pre	Post	Pre	Post	Pre	Post
250	360	0	0	400	400
500	600	250	240	0	80
0	0	0	0	0	0
400	480	300	280	400	500
480	480	0	0	200	200
480	480	0	0	80	120
400	480	240	240	0	0
120	100	250	360	220	200
0	0	220	240	180	180
350	400	150	160	220	240
500	450	0	0	0	0
0	0	280	280	150	180
410	400	0	0	800	720

	Obese		Obese + CI		Obese + CI + PA	
	Pre	Post	Pre	Post	Pre	Post
**Mean**	299.23	325.38	130.00	138.46	203.85	216.92
**SD**	200.89	217.43	129.81	140.11	226.92	212.11
**SE**	55.717	60.305	36.002	38.859	62.936	58.828

Average score of leisure-time physical activity (D) between the pre- and post-test within the same group

The average score of leisure-time physical activity between the pre- and post-test within the same group (D)					
Obese		Obese + CI		Obese + CI + PA	
Pre	Post	Pre	Post	Pre	Post
5000	5080	250	240	1200	2160
3000	3220	1500	1440	1500	1680
800	1360	0	0	1200	4400
2000	2160	0	0	1000	1200
1200	1080	200	200	700	1560
3000	2960	250	160	80	0
5500	5760	200	240	500	480
500	480	0	0	400	1120
1700	1680	0	0	700	1200
1200	1240	240	240	240	660
500	480	800	720	3800	6720
2000	2220	200	240	400	960
1500	1360	4500	3840	500	800

	Obese		Obese + CI		Obese + CI + PA	
	Pre	Post	Pre	Post	Pre	Post
**Mean**	2146.15	2236.92	626.15	563.08	940.00	1764.62
**SD**	1595.10	1641.76	1236.45	1061.13	955.13	1833.07
**SE**	442.402	455.343	342.930	294.304	264.904	508.402

The difference of total physical activity scores among the groups (E)

The difference of total physical activity scores among the groups (E)		
**Obese**	**Obese + CI**	**Obese + CI + PA**
100	180	780
-1700	320	1280
120	-160	1680
1600	220	1620
20	40	1620
120	-80	-80
520	-160	760
-250	240	640
380	-540	280
60	0	-220
-300	0	-100
-120	-10	920
-360	110	130

	Obese	Obese + CI	Obese + CI + PA
**Mean**	14.62	12.31	716.15
**SD**	718.87	223.95	685.83
**SE**	199.379	62.112	190.214

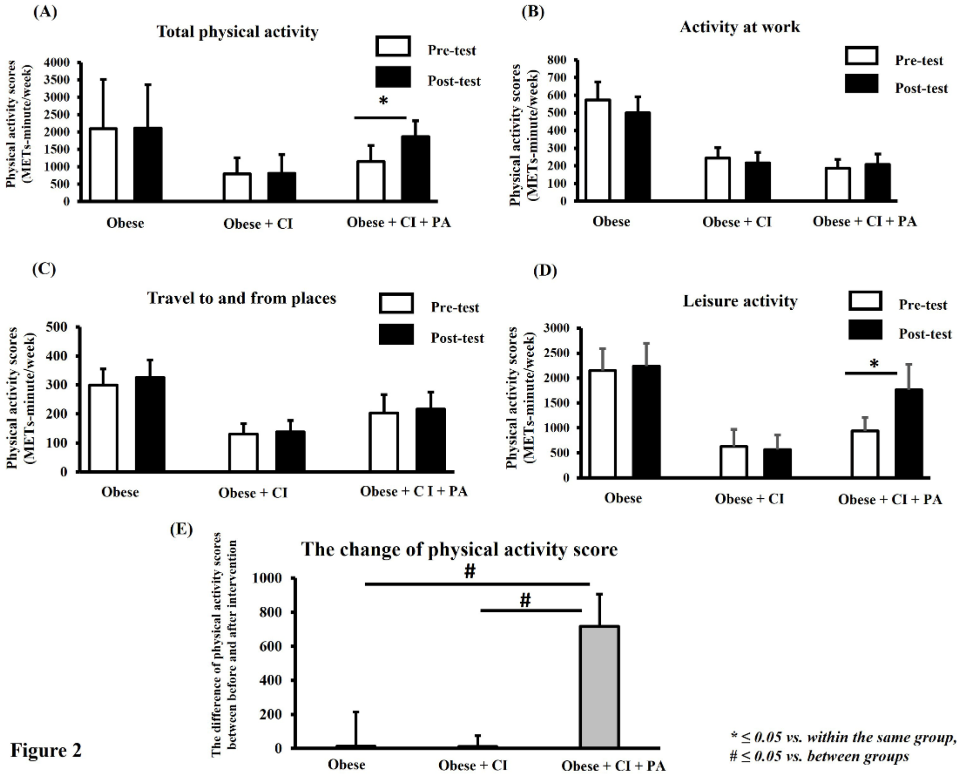

Figure 2
