# Supplementary material for: Social support-based physical activity that exerts beneficial effects for obese older adults with cognitive impairment via increasing participation in leisure-time physical activity
Source: PLoS One. 2025 Jun 30;20(6):e0325516. doi: 10.1371/journal.pone.0325516 (PMC12208442; doi:10.1371/journal.pone.0325516)
Supplement: S2 Fig — The results of physical functioning included arterial oxygen saturation (SPO2) (A), heart rate (B), and 6-MWT distance (6-MWD) (C) between the pre- and post-test within the same group. The difference of 6-MWD among the groups (D). The data set is shown below. (PDF) [file pone.0325516.s002.pdf]

S2 Fig. The physical functional capacity.

The results of physical functioning included arterial oxygen saturation (SPO2) (A), heart rate (B), and 6-MWT distance (6-MWD) (C) between the pre- and post-test within the same group. The difference of 6-MWD among the groups (D).

The arterial oxygen saturation (SPO2) (A)

The arterial oxygen saturation (SPO2) (A)					
Obese		Obese + CI		Obese + CI + PA	
Pre	Post	Pre	Post	Pre	Post
98	99	99	95	99	96
95	97	98	97	98	96
99	98	95	99	95	96
95	95	98	99	98	96
99	99	98	98	98	96
95	95	99	95	98	97
98	99	95	98	95	96
95	95	98	98	95	99
98	98	97	98	96	99
98	97	95	98	95	99
98	95	95	95	95	96
96	98	96	97	97	97
95	95	97	95	95	96

	Obese		Obese + CI		Obese + CI + PA	
	Pre	Post	Pre	Post	Pre	Post
**Mean**	96.85	96.92	96.92	97.08	96.46	96.85
**SD**	1.68	1.71	1.55	1.55	1.56	1.28
**SE**	0.465	0.473	0.431	0.431	0.433	0.355

The heart rate (B)

The heart rate (B)					
Obese		Obese + CI		Obese + CI + PA	
Pre	Post	Pre	Post	Pre	Post
89	84	83	85	88	77
88	95	80	89	82	78
85	94	82	80	86	80
88	89	88	88	87	81
88	92	82	80	85	79
88	87	86	85	83	76
85	88	87	88	86	78
85	86	85	85	87	74
86	88	83	84	88	75
87	87	83	80	84	76
88	88	83	85	80	75
84	82	85	87	80	77
86	84	83	84	75	78

	Obese		Obese + CI		Obese + CI + PA	
	Pre	Post	Pre	Post	Pre	Post
**Mean**	86.69	88.00	83.85	84.62	83.92	77.23
**SD**	1.60	3.83	2.23	3.07	3.84	2.05
**SE**	0.444	1.062	0.619	0.851	1.065	0.568

The 6-MWT distance (6-MWD) (C) between the pre- and post-test within the same group

The 6-MWT distance (6-MWD) (C) between the pre- and post-test within the same group					
Obese		Obese + CI		Obese + CI + PA	
Pre	Post	Pre	Post	Pre	Post
500	600	250	300	310	450
400	500	310	450	310	300
550	500	310	500	590	500
500	400	350	400	400	400
360	400	400	450	450	400
360	500	450	500	400	500
640	600	400	200	200	300
450	400	300	200	300	400
500	500	400	300	300	500
720	800	300	300	200	400
670	600	200	200	550	600
600	580	600	640	300	680
600	680	400	380	400	680

	Obese		Obese + CI		Obese + CI + PA	
	Pre	Post	Pre	Post	Pre	Post
**Mean**	526.92	543.08	359.23	370.77	362.31	470.00
**SD**	116.07	117.15	100.87	135.98	118.61	124.37
**SE**	32.193	32.490	27.976	37.715	32.898	34.493

The difference of 6-MWD among the groups (D)

The difference of 6-MWD among the groups (D)		
**Obese**	**Obese + CI**	**Obese + CI + PA**
100	50	140
100	140	-10
-50	190	-90
-100	50	0
40	50	-50
140	50	100
-40	-200	100
-50	-100	100
0	-100	200
80	0	200
-70	0	50
-20	40	380
80	-20	280

	Obese	Obese + CI	Obese + CI + PA
**Mean**	16.15	11.54	107.69
**SD**	77.73	102.46	133.80
**SE**	21.559	28.416	37.110

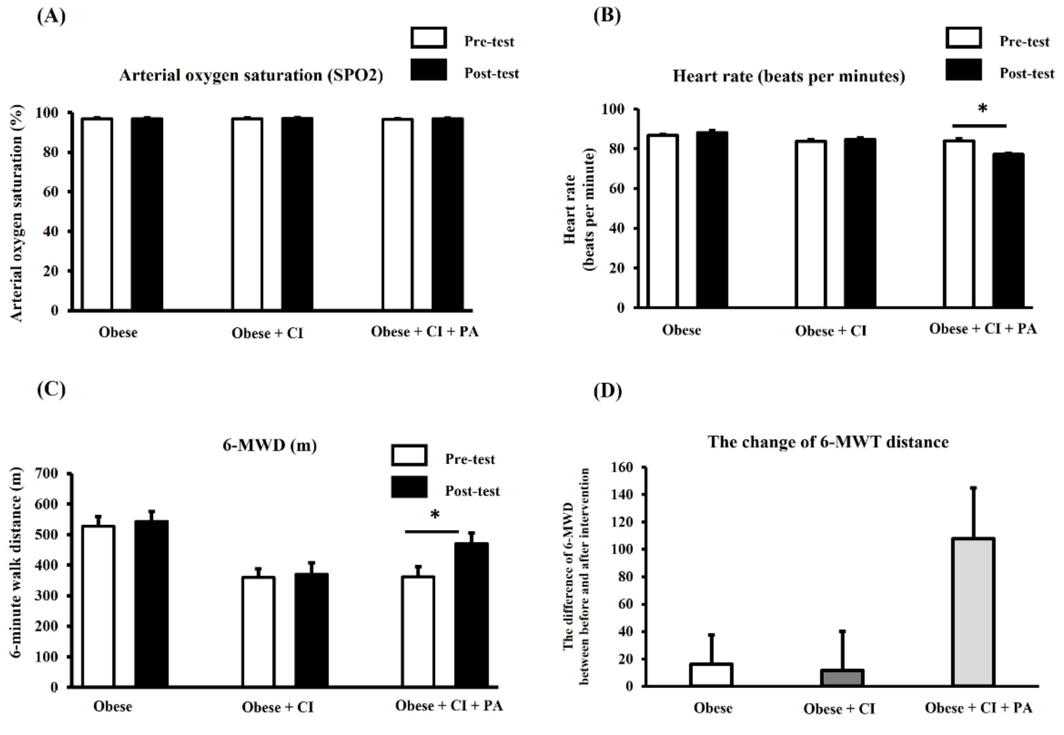

Figure 3

** ≤ 0.05 vs. within the same group*
