# Supplementary material for: Social support-based physical activity that exerts beneficial effects for obese older adults with cognitive impairment via increasing participation in leisure-time physical activity
Source: PLoS One. 2025 Jun 30;20(6):e0325516. doi: 10.1371/journal.pone.0325516 (PMC12208442; doi:10.1371/journal.pone.0325516)
Supplement: S3 Fig — The MoCA scores between the pre- and post-test within the same group (A). The difference of MoCA score among the groups (B). The data set is shown below. (PDF) [file pone.0325516.s003.pdf]

**S3 Fig. The level of cognitive function.**

The MoCA scores between the pre- and post-test within the same group (A). The difference of MoCA score among the groups (B).

**The MoCA scores between the pre- and post-test within the same group (A)**

| The MoCA scores between the pre- and post-test within the same group (A) | | | | | |
|---|---|---|---|---|---|
| Obese | | Obese + CI | | Obese + CI + PA | |
| Pre | Post | Pre | Post | Pre | Post |
| 24 | 27 | 6 | 7 | 15 | 20 |
| 26 | 27 | 21 | 22 | 13 | 18 |
| 26 | 26 | 23 | 21 | 15 | 20 |
| 22 | 24 | 17 | 18 | 20 | 25 |
| 26 | 27 | 19 | 20 | 12 | 17 |
| 25 | 27 | 13 | 15 | 11 | 16 |
| 25 | 24 | 12 | 14 | 14 | 18 |
| 23 | 26 | 11 | 12 | 12 | 17 |
| 29 | 29 | 13 | 12 | 17 | 22 |
| 24 | 25 | 15 | 16 | 12 | 17 |
| 25 | 25 | 22 | 23 | 22 | 24 |
| 27 | 26 | 16 | 17 | 17 | 22 |
| 25 | 24 | 19 | 18 | 18 | 23 |

| | Obese | | Obese + CI | | Obese + CI + PA | |
|---|---|---|---|---|---|---|
| | Pre | Post | Pre | Post | Pre | Post |
| **Mean** | 25.15 | 25.92 | 15.92 | 16.54 | 15.23 | 19.92 |
| **SD** | 1.77 | 1.50 | 4.91 | 4.56 | 3.39 | 3.01 |
| **SE** | 0.492 | 0.415 | 1.361 | 1.264 | 0.942 | 0.836 |

**The difference of MoCA score among the groups (B).**

| The difference of MoCA score among the groups (B) | | |
|---|---|---|
| **Obese** | **Obese + CI** | **Obese + CI + PA** |
| 3 | 1 | 5 |
| 1 | 1 | 5 |
| 0 | -2 | 5 |
| 2 | 1 | 5 |
| 1 | 1 | 5 |
| 2 | 2 | 5 |
| -1 | 2 | 4 |
| 3 | 1 | 5 |
| 0 | -1 | 5 |
| 1 | 1 | 5 |
| 0 | 1 | 2 |
| -1 | 1 | 5 |
| -1 | -1 | 5 |

| | Obese | Obese + CI | Obese + CI + PA |
|---|---|---|---|
| **Mean** | 0.77 | 0.62 | 4.69 |
| **SD** | 1.42 | 1.19 | 0.85 |
| **SE** | 0.395 | 0.331 | 0.237 |

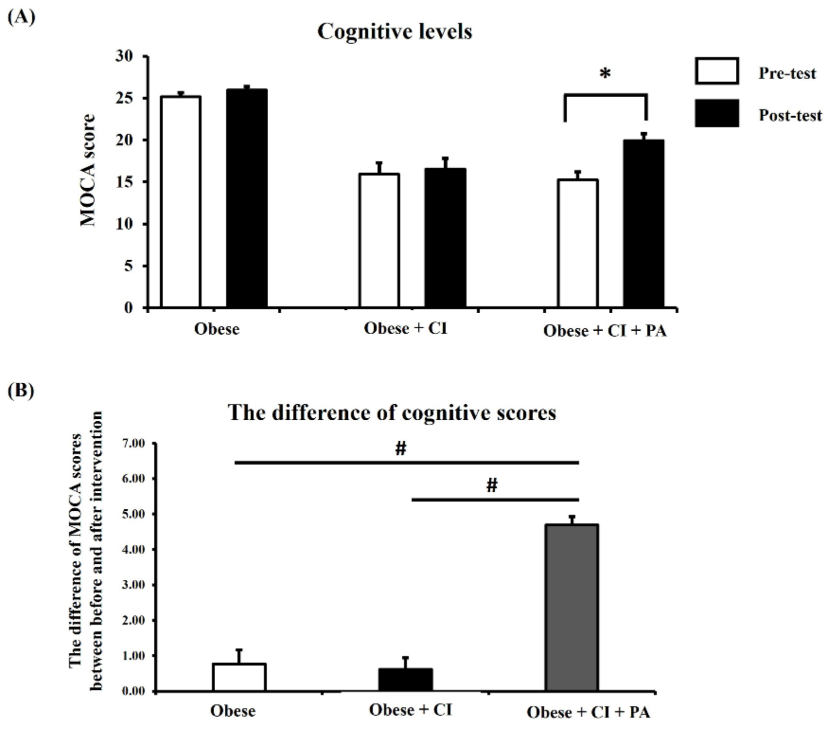


Figure 4

** ≤ 0.05 vs. within the same group, # ≤ 0.05 vs. between groups*
