# Supplementary material for: Social support-based physical activity that exerts beneficial effects for obese older adults with cognitive impairment via increasing participation in leisure-time physical activity
Source: PLoS One. 2025 Jun 30;20(6):e0325516. doi: 10.1371/journal.pone.0325516 (PMC12208442; doi:10.1371/journal.pone.0325516)
Supplement: S4 Fig — The PHQ-9 scores between the pre- and post-test within the same group (A). The difference of PHQ-9 scores among the groups (B). The data set is shown below. (PDF) [file pone.0325516.s004.pdf]

S4 Fig. The level of depression.

The PHQ-9 scores between the pre- and post-test within the same group (A). The difference of PHQ-9 scores among the groups (B).

The PHQ-9 scores between the pre- and post-test within the same group (A)

The PHQ-9 scores between the pre- and post-test within the same group (A)					
Obese		Obese + CI		Obese + CI + PA	
Pre	Post	Pre	Post	Pre	Post
2	2	6	5	5	3
0	0	4	6	6	3
1	1	6	6	6	1
0	0	5	5	5	2
1	1	9	10	7	2
0	0	6	6	6	3
0	0	5	5	5	1
1	1	6	5	6	4
2	2	8	9	8	5
1	1	9	9	5	1
1	2	9	9	9	3
2	2	6	5	7	4
2	2	6	6	6	4

	Obese		Obese + CI		Obese + CI + PA	
	Pre	Post	Pre	Post	Pre	Post
**Mean**	1.00	1.08	6.54	6.62	6.23	2.77
**SD**	0.82	0.86	1.66	1.89	1.24	1.30
**SE**	0.226	0.239	0.462	0.525	0.343	0.361

The difference of PHQ-9 scores among the groups (B).

The difference of PHQ-9 scores among the groups (B)		
**Obese**	**Obese + CI**	**Obese + CI + PA**
0	-1	-2
0	2	-3
0	0	-5
0	0	-3
0	1	-5
0	0	-3
0	0	-4
0	-1	-2
0	1	-3
0	0	-4
1	0	-6
0	-1	-3
0	0	-2

	Obese	Obese + CI	Obese + CI + PA
**Mean**	0.08	0.08	-3.46
**SD**	0.28	0.86	1.27
**SE**	0.077	0.239	0.351

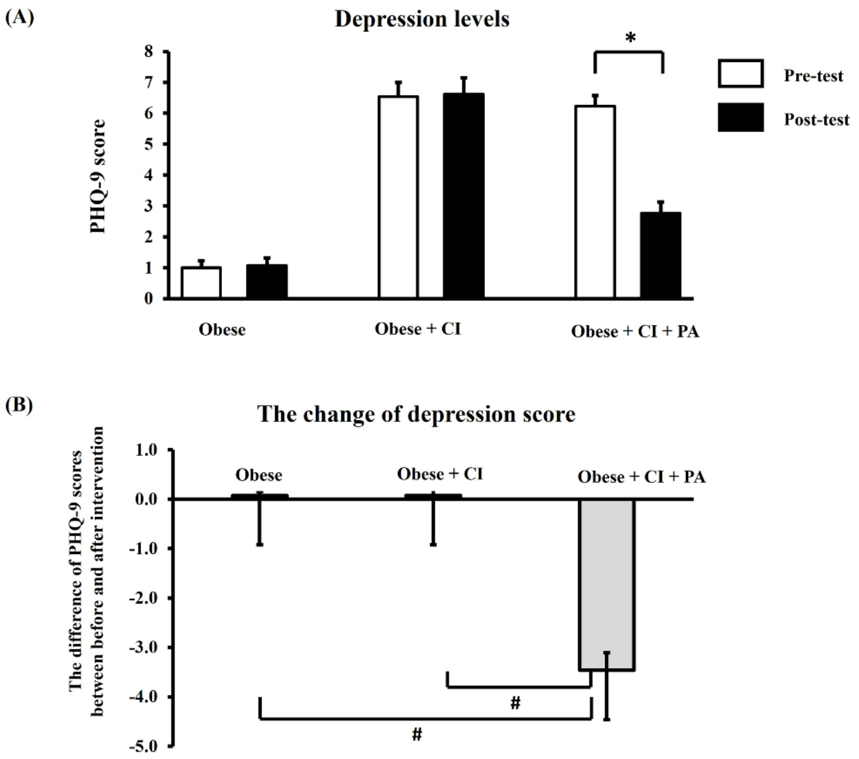

Figure 5

** ≤ 0.05 vs. within the same group, # ≤ 0.05 vs. between groups*
